# Direct Measurement of Adipose Thermogenesis by Isothermal Microcalorimetry

**DOI:** 10.3390/cells15070579

**Published:** 2026-03-25

**Authors:** Pauke C. Schots, Devesh Kesharwani, Chad C. Doucette, Aaron C. Brown

**Affiliations:** 1 Faculty of Biosciences, Norwegian College of Fishery Science, Fisheries and Economics, UiT-The Arctic University of Norway, 9037 Tromsø, Norway; pauke.schots@uit.no; 2 Center for Molecular Medicine, Maine Medical Center Research Institute, 81 Research Drive, Scarborough, ME 04074, USA; devesh.kesharwani@mainehealth.org (D.K.); chad.doucette@mainehealth.org (C.C.D.); 3 School of Biomedical Sciences and Engineering, The University of Maine, Orono, ME 04469, USA; 4 Tufts University School of Medicine, 145 Harrison Avenue, Boston, MA 02111, USA

**Keywords:** adipose tissue, thermogenesis, isothermal microcalorimetry, brown adipose tissue, beige adipocytes, energy expenditure, ex vivo metabolism

## Abstract

Direct measurement of thermogenic heat production remains a major challenge in adipose biology. Isothermal microcalorimetry offers a label-free approach to quantify metabolic heat output, yet key parameters governing its application to adipose tissue remain poorly defined. Here, we systematically evaluate the use of the CalScreener isothermal microcalorimetry platform to quantify thermogenic heat production across multiple adipose models, including adipocyte spheroids, freshly isolated adipocytes, and intact adipose tissue explants. Heat production scaled with spheroid size within a defined range and increased linearly with spheroid number per well, demonstrating the quantitative sensitivity of the calorimetric measurements. Pharmacological modulation of mitochondrial respiration in cultured primary beige adipocytes demonstrated that oxidative phosphorylation is a major driver of the calorimetric heat signal, including heat generation associated with mitochondrial proton leak. Freshly isolated adipocytes and intact adipose tissue exhibited depot-specific thermogenic activity and retained responsiveness to β-adrenergic stimulation ex vivo. Across adipose depots, intact tissue explants revealed unexpected differences in thermogenic heat production that were not fully reflected by thermogenic gene expression, highlighting divergence between molecular and functional readouts. Intact adipose tissue maintained measurable thermogenic heat production following extended ex vivo handling in nutrient-containing medium, such that tissues collected across a prolonged harvest window exhibited comparable calorimetric activity, enabling batch analysis of large experimental cohorts. Microcalorimetry further resolved regional differences in thermogenic heat production within the inguinal adipose depot following cold exposure. Together, these findings define key experimental considerations for applying isothermal microcalorimetry to adipose biology and demonstrate its utility for directly quantifying thermogenic metabolism in cells and intact tissues.

## 1. Introduction

Obesity is a major global health challenge that increases the risk of metabolic disorders including type 2 diabetes, cardiovascular disease, metabolic-associated steatotic liver disease and cancer [1,2,3]. At a physiological level, obesity reflects a chronic imbalance between energy intake and energy expenditure that is driven in part by dysfunction of adipose tissue, a central regulator of systemic metabolic homeostasis [4]. While lifestyle modification remains the cornerstone of obesity management, long-term efficacy is limited. Although pharmacological and surgical interventions can be effective, they are costly, associated with adverse effects, and frequently followed by weight regain [5]. These limitations have motivated increased interest in strategies that enhance energy expenditure rather than restricting energy intake.

Thermogenic adipocytes, including brown adipocytes and inducible beige adipocytes within white adipose tissue, dissipate chemical energy as heat and play a critical role in metabolic regulation [6,7,8]. Brown adipocytes reside in dedicated depots and are specialized for constitutive thermogenesis, whereas beige adipocytes emerge within subcutaneous white adipose tissue in response to environmental cues such as cold exposure [7]. Thermogenic activation is driven largely by sympathetic signaling through beta-adrenergic receptors, leading to increased substrate uptake, mitochondrial activity, and heat production [9,10]. Although uncoupling protein 1 (UCP1) is a central mediator of thermogenesis, additional *UCP1*-independent mechanisms, including calcium cycling, creatine turnover, and futile lipid cycling, also contribute to energy dissipation [11].

Despite the recognized importance of thermogenic adipocytes, accurately quantifying thermogenic activity remains technically challenging. Traditional approaches rely on surrogate measures such as gene and protein expression, oxygen consumption assays using Seahorse or Oroboros platforms, or glucose uptake assessed by fluorine-18 fluorodeoxyglucose positron emission tomography (PET) imaging [12,13,14]. While informative, these methods do not directly measure heat production, which is the defining functional output of thermogenesis. Recent advances in isothermal microcalorimetry have enabled direct, label-free measurement of heat production in living cells and tissues, providing an integrated readout of metabolic activity. Prior studies have demonstrated the feasibility of using the CalScreener isothermal microcalorimeter to quantify heat production in isolated primary and cell line-derived adipocytes, highlighting the potential of this approach for studying adipose tissue metabolism [15,16]. However, several experimental considerations governing the broader application of isothermal microcalorimetry to adipose biology remain poorly defined. These include optimal sample configurations, such as spheroid size and number, the stability of thermogenic activity in intact adipose tissue ex vivo, and the impact of tissue handling and storage conditions on calorimetric measurements. In particular, it remains unclear how long intact adipose tissue can retain functional thermogenic activity outside the organism, which is an important practical consideration for experiments requiring extended tissue harvest times or batch analysis of large experimental cohorts.

In this study, we evaluate the application of isothermal microcalorimetry to measure thermogenesis across multiple adipose models, including adipocyte spheroids, freshly isolated adipocytes, and intact adipose tissue explants from distinct depots. We demonstrate quantitative scaling of calorimetric heat production with spheroid number, show that calorimetric heat output reflects mitochondrial respiration in thermogenic adipocytes, and assess thermogenic activity in intact adipose tissue ex vivo. We further examine depot-specific differences in heat production and determine how tissue handling and storage conditions influence calorimetric measurements. Together, these findings provide guidance for applying isothermal microcalorimetry to the study of adipose thermogenesis across cellular and tissue models.

## 2. Methods

### 2.1. Reagents and Buffers

BIOPS (biopsy preservation solution) buffer was prepared as a physiological preservation buffer containing 10 mM Ca-EGTA buffer (0.1 µM free Ca^2+^), 20 mM imidazole, 20 mM taurine, 50 mM K-MES, 0.5 mM dithiothreitol (DTT), 6.56 mM MgCl_2_, 5.77 mM ATP, and 15 mM phosphocreatine, adjusted to pH 7.1. Dulbecco’s modified Eagle’s medium (DMEM; high glucose) supplemented with GlutaMAX™ and HEPES (11965-092, 35050061 and 32430027, Gibco™, Waltham, MA, USA) and 1% bovine serum albumin (BSA; fraction V) was used for tissue incubation and calorimetric measurements. CL316243 was purchased from Cayman Chemical (17499, Ann Arbor, MI, USA). Isoproterenol hydrochloride was purchased from Sigma-Aldrich (I5627, Saint Louis, MO, USA). Oligomycin (2.5 µM), FCCP (2.5 µM), antimycin A (2 µM) and rotenone (2 µM) were used for pharmacological inhibitors of mitochondrial respiration.

### 2.2. Animal Ethics and Husbandry

All animal studies were conducted in accordance with applicable national and institutional guidelines and were approved by the relevant animal ethics committees at each study site. Experiments performed in the United States were conducted in compliance with the National Institutes of Health Guide for the Care and Use of Laboratory Animals and were approved by the Institutional Animal Care and Use Committee (IACUC; protocol #2507) at the MaineHealth Institute for Research (MHIR). Experiments performed in Norway were approved by the institutional animal use authority at the Faculty of Health Sciences, UiT–The Arctic University of Norway (approval reference AKM 04/25) and were conducted in accordance with Directive 2010/63/EU on the protection of animals used for scientific purposes.

All mice used in this study were adult C57BL/6 animals aged 8–11 weeks. Mice were housed in pathogen-free facilities under controlled environmental conditions with a 12 h light/dark cycle and ad libitum access to food and water. Animals euthanized at MHIR were sacrificed by carbon dioxide (CO_2_) inhalation, whereas animals euthanized at UiT were sacrificed by pentobarbital overdose. All efforts were made to minimize animal suffering and reduce animal numbers.

### 2.3. In Vitro Adipocyte Culture and Differentiation

Three-dimensional adipocyte spheroids were generated using attachment-free spheroid culture plates (Greiner Bio-One CELLSTAR 96-well microplate, 650979, Monroe, NC, USA). Immortalized interscapular brown (ThermoMouse) and mouse inguinal adipose tissue derived beige (Cat# EVC005, Kerafast, Inc., Boston, MA, USA) preadipocyte cell lines were seeded at defined cell numbers per well and allowed to self-assemble into spheroids prior to differentiation [17,18]. Cells were then induced to differentiate using a defined adipogenic cocktail containing insulin, triiodothyronine (T3), rosiglitazone, IBMX, dexamethasone, indomethacin, SB431542, and ascorbic acid phosphate as previously described [16,19]. After 3 days, cultures were transitioned to maintenance medium containing insulin, T3, rosiglitazone, SB431542, and ascorbic acid phosphate for 3 days, and were maintained until day 10 of differentiation in basal medium (DMEM + 10% FBS) prior to CalScreener analysis.

### 2.4. Isolation of Mature Adipocytes

Mature adipocytes were isolated from interscapular brown, inguinal white and gonadal white adipose tissue of adult mice. Dissected adipose tissues were minced and digested at 37 °C for 30–40 min in tissue lysis buffer containing 0.123 M NaCl, 1.3 mM CaCl_2_, 5 mM glucose, 100 mM HEPES, 4% (*v*/*v*) bovine serum albumin (BSA; fraction V), and 0.1% (*w*/*v*) Collagenase P with constant rocking. Following digestion, an equal volume of autoMACS^®^ Running Buffer (130-091-221, Waltham, MA, USA) was added to each sample to inhibit the collagenase activity. Tissue lysates were then allowed to stand at room temperature for 10 min to facilitate separation of mature adipocytes, which floated to the upper fraction. Mature adipocytes were carefully collected from the floating layer using a 22-gauge syringe and washed with sterile autoMACS^®^ Running Buffer to remove residual debris and stromal cells. Cell viability and concentration were determined using the Cellometer K2 Fluorescent Cell Counter (Revvity, Inc., Waltham MA, USA) following staining with ViaStain™ AOPI solution (CS2-0106-5ML, Revvity, Inc.), according to the manufacturer’s instructions.

### 2.5. Adipose Tissue Dissection and Preparation

Interscapular BAT, inguinal WAT, and gonadal WAT were rapidly dissected, cleaned of visible connective tissue, and sectioned using sterile scalpel blades. Tissue sections were weighed prior to incubation in the Calscreener. Typical BAT sections ranged from approximately 4 to 22 mg, whereas WAT sections ranged from approximately 40 to 175 mg depending on depot. Tissue sections were immediately placed into DMEM or BIOPS buffer depending on the experimental condition.

### 2.6. Temporary Storage of Adipose Tissue

To evaluate the effects of short-term storage prior to calorimetric analysis, adipose tissue sections were stored for up to 6 h under defined conditions. Storage conditions included incubation in nutrient-containing DMEM at 37 °C or storage on ice in either DMEM or BIOPS buffer. Tissues assigned to storage conditions were harvested at the beginning of the storage period, whereas freshly isolated control tissues were harvested near the end of the same time window.

All tissues were loaded into the CalScreener simultaneously to allow direct comparison. For calorimetric measurements, all tissues, including those previously stored in BIOPS, were transferred into DMEM prior to loading to ensure equivalent nutrient availability during measurement.

### 2.7. Isothermal Microcalorimetry

The CalScreener (Symcel AB, Solna, Sweden) is a highly sensitive isothermal microcalorimetry platform used to measure metabolic heat production from living cells or tissues in real time. Each sealed vial is equipped with highly sensitive thermopile sensors that detect extremely small temperature differences generated by biological heat production. The principle underlying CalScreener measurements is based on isothermal microcalorimetry, which quantifies the heat released during cellular metabolic processes. As cells perform biochemical reactions- including mitochondrial respiration, substrate oxidation, and ATP turnover- they release heat as a by-product of metabolism. The calorimeter detects these heat flux changes and converts them into a continuous signal representing metabolic power (μW). Because heat generation is directly proportional to overall metabolic activity, microcalorimetry provides a label-free and non-invasive method to assess cellular energy expenditure and thermogenic capacity.

Heat production was measured using a 48 well isothermal microcalorimeter plate. Plastic inserts were filled with 200 µL DMEM, adjusted to account for tissue volume by assuming a tissue density of 1 mg/µL to maintain comparable oxygen availability across samples. Tissue sections, isolated adipocytes, or adipocyte spheroids were placed into the inserts, which were then sealed within titanium calVial™ capsules and loaded into the calPlate™ holder. When indicated, CL316243 (2 µM) or isoproterenol (500 µM) was added immediately prior to sealing the vials. For pharmacological mitochondrial oxidative phosphorylation inhibition, oligomycin (2.5 µM), FCCP (2.5 µM), antimycin A (2 µM) and rotenone (2 µM) were added to calscreener vial prior to incubation. After insertion into the instrument, samples underwent a 30 min equilibration and calibration period, followed by an additional 15 min stabilization period before continuous heat flow was recorded. Accordingly, heat measurements reflect metabolic activity beginning approximately 45 min after sample preparation and addition of pharmacological treatments. This timing was consistent across all experimental conditions and did not differentially affect comparisons between groups. Measurements were performed for 4–16 h depending on the experimental condition. In all microcalorimetry experiments, medium-filled crucibles were used as controls to match thermal inertia and heat capacity between control and sample conditions and minimize heat-buffering artifacts. Control crucibles contained the corresponding culture medium without biological material and were applied consistently across experiments with isolated cells, spheroids, and adipose tissue explants. Baseline heat signals were subtracted to ensure that measured heat flux reflected metabolic heat production.

### 2.8. Statistical Analysis

All data are presented as mean ± standard error of the mean (SEM). Comparisons between two groups were performed using unpaired two-tailed Student’s *t*-tests. For comparisons involving more than two groups, one-way analysis of variance (ANOVA) and two-way analysis of variance (ANOVA) were used followed by Tukey’s multiple-comparison test and Fisher’s LSD test, respectively, where appropriate. A *p*-value < 0.05 was considered statistically significant. Significance levels are indicated as follows: ns, not significant; * *p* < 0.05, ** *p* < 0.01, *** *p* < 0.001, **** *p* < 0.0001.

## 3. Results

### 3.1. Optimization of Adipocyte Spheroid Size and Number for Calorimetric Measurement of Thermogenesis

**Figure 1:**
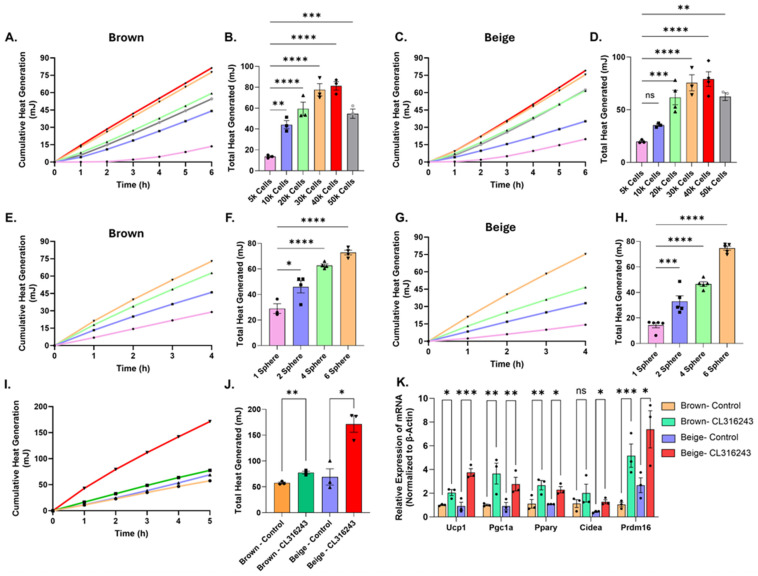
Quantitative measurement of thermogenic heat production using adipocyte spheroids. (**A**) Cumulative heat production over time from differentiated brown adipocyte spheroids generated from 5000 to 50,000 preadipocytes per spheroid. (**B**) Total heat production from brown adipocyte spheroids of increasing size (*n* = 3). (**C**) Cumulative heat production over time from differentiated beige adipocyte spheroids generated across the same range of initial cell numbers. (**D**) Total heat production from beige adipocyte spheroids of increasing size (*n* = 3). (**E**) Cumulative heat production over time from brown adipocyte spheroids with increasing numbers of spheroids (1–6) per CalWell. (**F**) Total heat production from brown adipocyte spheroids as a function of spheroid number per well (*n* = 3–4). (**G**) Cumulative heat production over time from beige adipocyte spheroids with increasing numbers of spheroids per CalWell. (**H**) Total heat production from beige adipocyte spheroids as a function of spheroid number per well (*n* = 5). (**I**) Cumulative heat production over time from pooled brown and beige adipocyte spheroids treated with vehicle or the β3-adrenergic agonist CL316243. (**J**) Total heat production from brown and beige adipocyte spheroids following vehicle or CL316243 treatment (*n* = 3). (**K**) qPCR analysis of thermogenic gene expression in brown and beige adipocyte spheroids with or without CL316243 treatment after 6 h (*n* = 3). Data are shown as mean ± SEM. Individual data points represent independent CalWell and qPCR measurements. Statistical analyses were performed using one-way ANOVA (**B**,**D**,**F**,**H**) and two-way ANOVA (**J**,**K**) as indicated. Significance levels are indicated as follows: ns, not significant; * *p* < 0.05, ** *p* < 0.01, *** *p* < 0.001, **** *p* < 0.0001. Line colors and data point symbols in the cumulative heat generation plots correspond to those used in the adjacent total heat generation bar graphs.

We first assessed the sensitivity of the CalScreener to detect heat production from three-dimensional adipocyte spheroids, which allow precise control over total cell number compared to intact adipose tissue explants. Brown adipocyte spheroids were generated using attachment-free wells by seeding between 5000 and 50,000 mouse preadipocytes from an immortalized ThermoMouse-derived cell line per spheroid, followed by differentiation into mature brown adipocytes [17]. Individual spheroids were loaded into CalWell vessels with three technical replicates per spheroid size. Heat production increased as the number of cells per spheroid increased from 5000 to 30,000 cells, plateaued between 30,000 and 40,000 cells, and declined at 50,000 cells (Figure 1A,B). A similar size-dependent relationship was observed using a separate immortalized beige preadipocyte cell line after differentiation into mature adipocytes (Figure 1C,D).

We next examined whether increasing the number of spheroids per CalWell produced proportional increases in calorimetric heat output. Spheroids seeded with 30,000 preadipocytes were differentiated into either brown or beige adipocytes, and between one and six spheroids were added per CalWell (Figure 1E–H). Heat production increased linearly with spheroid number for both brown and beige adipocyte spheroids, demonstrating quantitative detection of thermogenic heat production by the calorimetric platform. Wells containing four to six spheroids also exhibited reduced variability across replicate wells compared with wells containing one or two spheroids.

To confirm adipogenic differentiation across spheroid sizes, qPCR analysis of adipogenic markers (*Pparγ* and *Cebpa*) was performed (Supplementary Figure S1A–D). In brown adipocyte spheroids, *Pparγ* and *Cebpa* expression were significantly elevated in spheroids generated with 30,000–50,000 cells. In contrast, beige adipocyte spheroids exhibited maximal adipogenic gene expression at 30,000 cells, suggesting an optimal cell density for beige adipocyte differentiation. Consistent with these findings, immunostaining and H&E analysis of beige adipocyte spheroids demonstrated increased adipocyte differentiation and spheroid size between 5000 and 30,000 cells. Spheroids generated with higher cell numbers (40,000–50,000 cells) displayed reduced adipocyte differentiation and lower cellular density in the spheroid core (Supplementary Figure S2).

Although cell viability or oxygen gradients were not directly measured, reduced cellular density in the cores of larger spheroids is consistent with previously described diffusion limitations in three-dimensional spheroid systems [20,21]. H&E staining further revealed decreased cellular density and increased extracellular matrix deposition in the central regions of larger spheroids (Supplementary Figure S2). Despite the decline in total heat output at the largest spheroid sizes, cumulative heat production increased approximately linearly over the 6 h measurement period for all spheroid sizes, including those seeded with 50,000 cells, suggesting that gross medium depletion was unlikely to limit metabolic activity during the assay (Figure 1A,C).

To determine whether adipocyte spheroids remained functionally thermogenic, pooled spheroids were treated with the β3-adrenergic agonist CL316243. Both brown and beige adipocyte spheroids exhibited increased heat production following stimulation (Figure 1I–J). Basal heat output was similar between brown and beige adipocyte spheroids derived from the selected cell lines; however, beige adipocytes exhibited a stronger thermogenic response to CL316243. qPCR analysis revealed no significant difference in basal *Ucp1* expression between brown and beige spheroids, consistent with the comparable basal heat production measured by microcalorimetry. Upon CL316243 stimulation, *Ucp1* and other thermogenic genes were significantly upregulated in both brown and beige spheroids, with greater induction observed in beige adipocytes (Figure 1K). These findings indicate that adipocyte spheroids remain functionally responsive to β3-adrenergic stimulation.

Together, these results demonstrate that the CalScreener detects thermogenic heat production from adipocytes organized in three-dimensional spheroids across a defined range of cell numbers. Excessively large spheroids exhibited reduced heat output, while increasing the number of spheroids per well produced a proportional increase in calorimetric signal. These findings define key experimental parameters for applying adipocyte spheroids in calorimetric measurements of thermogenic activity.

### 3.2. Direct Measurement of Sustained Thermogenic Heat Production in Freshly Isolated Adipocytes

We next performed ex vivo studies to determine whether heat production from adipocytes freshly isolated from mouse adipose tissue could be directly measured and whether these cells retained responsiveness to thermogenic stimulation. Interscapular brown (iBAT) and subcutaneous inguinal (iWAT) adipose depots were pooled from multiple mice and enzymatically digested to isolate mature adipocytes. The floating adipocyte fraction was collected, and equivalent numbers of adipocytes were loaded per well for microcalorimetric analysis using the CalScreener. Heat production values were then normalized to the number of viable adipocytes to enable comparison between depots. Heat measurements reflect metabolic activity beginning approximately 45 min after sample preparation and addition of stimuli, following instrument equilibration and stabilization, and this timing was consistent across all experimental conditions. Cell viability was assessed prior to analysis using acridine orange/propidium iodide (AOPI) staining, and comparable viability was observed between adipocytes isolated from iBAT and iWAT depots. We note that adipocytes from different depots exhibit distinct morphological characteristics, with white adipocytes generally larger and unilocular compared to smaller, multilocular brown adipocytes. These differences, together with the inherent fragility of mature adipocytes during isolation, may introduce variability and influence calorimetric measurements when normalized on a per-cell basis.

**Figure 2:**
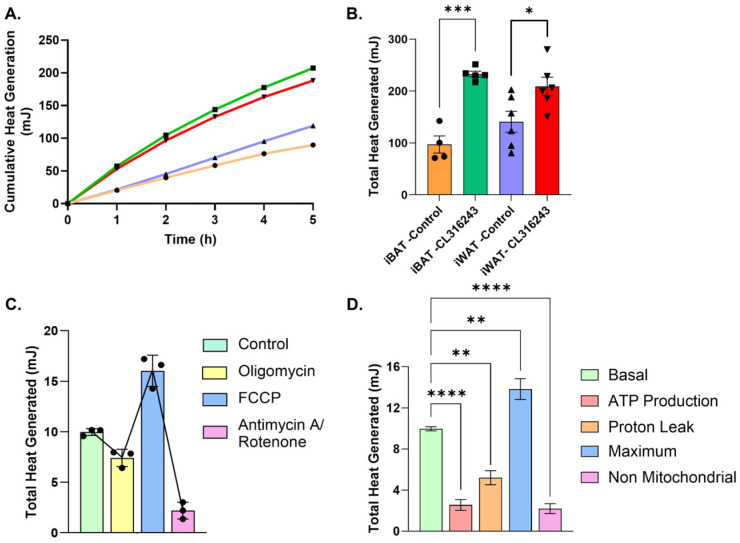
Direct measurement of sustained thermogenic heat production in freshly isolated adipocytes. (**A**) Cumulative heat production over time from mature adipocytes freshly isolated from interscapular brown adipose tissue (iBAT) (*n* = 4–5) or subcutaneous inguinal adipose tissue (iWAT) (*n* = 6) and treated with vehicle or the β3-adrenergic agonist CL316243. (**B**) Total heat production from freshly isolated brown and inguinal adipocytes following vehicle or CL316243 treatment. (**C**) Total heat production from cultured primary beige adipocytes following treatment with pharmacological modulators of mitochondrial respiration (oligomycin, FCCP, and antimycin A/rotenone) (*n* = 3). (**D**) Heat production associated with distinct phases of mitochondrial respiration, including ATP-linked respiration, proton leak, maximal respiration, and non-mitochondrial respiration, in cultured primary beige adipocytes. Data are shown as mean ± SEM. Individual data points represent independent CalWell measurements. Statistical analyses were performed using unpaired Student’s *t*-test (**B**) and one-way ANOVA (**D**) as indicated. Significance levels are indicated as follows: * *p* < 0.05, ** *p* < 0.01, *** *p* < 0.001, **** *p* < 0.0001. Line colors and data point symbols in the cumulative heat generation plots correspond to those used in the adjacent total heat generation bar graphs.

Under basal conditions, heat production did not differ significantly between isolated iBAT and iWAT adipocytes, although greater variability was observed in iWAT samples. Mature brown adipocytes exhibited a ~140% increase in heat production in response to the β3-adrenergic agonist CL316243, with heat output continuing to rise throughout the 6 h measurement period (Figure 2A,B). Inguinal adipocyte-derived cells showed a more modest but significant ~50% increase in heat production that also increased over time (Figure 2A,B). These findings indicate that adipocytes freshly isolated from mouse adipose tissue remain viable and capable of mounting sustained thermogenic responses ex vivo over the duration of tissue processing and calorimetric measurement.

To assess the contribution of mitochondrial oxidative phosphorylation to heat production, we performed additional experiments using pharmacological inhibitors of mitochondrial respiration (oligomycin, FCCP, and antimycin A/rotenone) in cultured primary beige adipocytes (Figure 2C). Oligomycin and antimycin A/rotenone significantly reduced heat production, whereas FCCP increased heat output, consistent with known effects on mitochondrial respiration and proton leak. Analysis of heat output across distinct phases of mitochondrial respiration, including ATP-linked respiration, proton leak, maximal respiration, and non-mitochondrial respiration, revealed significant changes in heat generation relative to basal conditions (Figure 2D). Together, these results demonstrate that calorimetric heat output reflects mitochondrial respiration and confirm that thermogenic heat production in adipocytes is predominantly driven by mitochondrial activity.

### 3.3. Depot-Specific Differences in Thermogenic Heat Production in Adipose Tissue Explants

**Figure 3:**
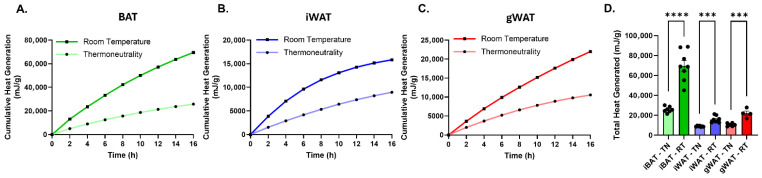
Depot-specific thermogenic heat production in intact adipose tissue explants. (**A**–**C**) Cumulative heat production over time, normalized per gram of tissue, measured by isothermal microcalorimetry from freshly isolated intact adipose tissue explants of iBAT (**A**), iWAT (**B**), and gWAT (**C**) harvested from mice housed at room temperature or thermoneutrality. (**D**) Total heat production per gram of tissue from iBAT (*n* = 7–8), iWAT (*n* = 7–8), and gWAT (*n* = 4–6) explants under each condition. Data are shown as mean ± SEM. Individual data points represent independent tissue explants. Statistical significance was assessed using an unpaired Student’s *t*-test as indicated. Tissue mass per well ranged from approximately 4–22 mg for iBAT and 40–175 mg for white adipose depots. Significance levels are indicated as follows: *** *p* < 0.001, **** *p* < 0.0001. Line colors and data point symbols in the cumulative heat generation plots correspond to those used in the adjacent total heat generation bar graphs.

We next assessed thermogenic heat production in freshly isolated intact adipose tissue explants using microcalorimetry over a 16 h period. Adipose depots were rapidly harvested and immediately placed in DMEM prior to analysis. Interscapular brown adipose tissue (iBAT) exhibited an approximately 350% increase in heat output per gram of tissue compared with subcutaneous inguinal adipose tissue (iWAT) and gonadal adipose tissue (gWAT), which produced comparable but lower levels of heat (Figure 3) at thermoneutral temperature. When mice were housed at thermoneutrality for 7 days prior to tissue harvest, heat production was significantly reduced across all depots, although the relative pattern was preserved (iBAT > iWAT ≈ gWAT) (Figure 3). iBAT exhibited the largest reduction, with heat production decreased by approximately 70% compared to room temperature-housed mice, whereas iWAT and gWAT each showed approximately 50% reductions. Unexpectedly, gWAT consistently exhibited heat production equivalent to or slightly higher than iWAT, despite the established association of subcutaneous depots with greater browning potential [22,23].

To further evaluate depot-specific responses, wild-type mice were housed at thermoneutrality (29 °C) for 10 days to minimize basal thermogenic activity, followed by cold exposure (6.5 °C) for 3 days (Supplementary Figure S3A). Under thermoneutral conditions, microcalorimetry analysis revealed comparable heat production between gWAT and iWAT explants (Supplementary Figure S3B,C). Following cold exposure, gWAT explants exhibited significantly higher heat production than iWAT (Supplementary Figure S3B,C). In contrast, qPCR analysis demonstrated a significant induction of *Ucp1* expression in iWAT relative to gWAT after cold exposure (Supplementary Figure S3D), indicating that canonical thermogenic gene activation is more robust in iWAT. These findings highlight a divergence between thermogenic gene expression and calorimetric heat output across adipose depots.

Analysis of heat production kinetics at room temperature revealed that both depots displayed similar initial heat generation rates; however, heat output in iWAT plateaued over the 16 h measurement period, whereas gWAT maintained a sustained increase in heat production (Figure 3A–C). Previous studies have shown that full browning of iWAT typically requires prolonged cold exposure (~7–10 days), raising the possibility that the 3-day cold challenge used here may not fully capture the maximal thermogenic capacity of iWAT [24]. Together, these findings suggest that differences in the temporal dynamics of thermogenic activation or the ability to sustain metabolic activity ex vivo may contribute to the higher cumulative heat production observed in gWAT under these conditions.

### 3.4. Regional Differences in Thermogenic Heat Production Within Inguinal Adipose Tissue Emerge Following Cold Exposure

The iWAT depot has been reported to exhibit spatial heterogeneity in thermogenic potential and *Ucp1* expression along its longitudinal axis during browning [25,26]. To determine whether regional differences in thermogenic heat production could be detected ex vivo, iWAT depots were sectioned longitudinally and transversely to generate anatomically distinct subsections (Supplementary Figure S4A). Under room temperature conditions, no significant differences in heat production were observed between left and right longitudinal sections or between upper and lower transverse sections of the depot (Supplementary Figure S4B,C), indicating relatively uniform thermogenic activity across the iWAT depot under basal conditions.

**Figure 4:**
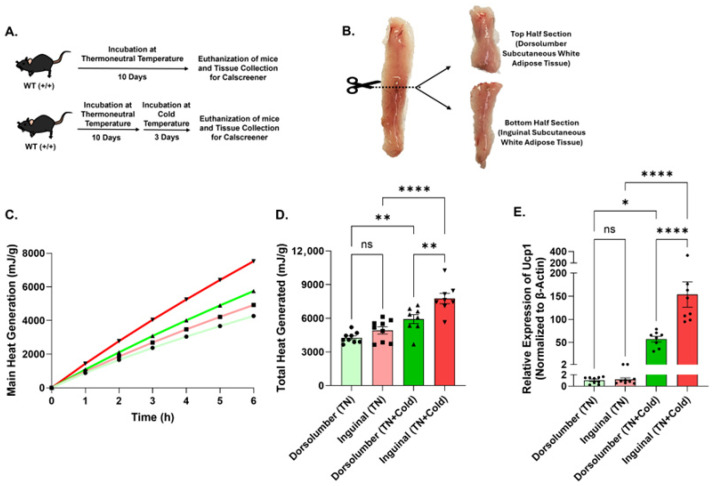
Regional differences in thermogenic heat production within inguinal adipose tissue. (**A**) Schematic illustration of wild-type mice housed under thermoneutral (TN) and cold exposure conditions. (**B**) Schematic representation of iWAT sectioning for microcalorimetric analysis using the CalScreener. (**C**) Cumulative heat production over time, normalized per gram of tissue, measured by isothermal microcalorimetry from ex vivo iWAT subsections corresponding to dorsolumbar (top) and inguinal (bottom) regions. (**D**) Total heat production per gram of tissue from each iWAT subsection (*n* = 8–9). (**E**) qPCR analysis of *Ucp1* expression in iWAT subsections (*n* = 8–9). Data are shown as mean ± SEM. Individual data points represent independent tissue sections. Statistical analyses were performed using one-way ANOVA as indicated. Significance levels are indicated as follows: ns, not significant; * *p* < 0.05, ** *p* < 0.01, **** *p* < 0.0001. Line colors and data point symbols in the cumulative heat generation plots correspond to those used in the adjacent total heat generation bar graphs.

To assess whether thermogenic stimulation reveals regional differences, wild-type mice were housed at thermoneutrality (29 °C) for 10 days to minimize baseline thermogenic activity and subsequently exposed to cold (6.5 °C) for 3 days to activate thermogenic programs in beige adipocytes (Figure 4A). iWAT depots were harvested from thermoneutral (TN) and cold-exposed (TN + Cold) mice and sectioned using the lymph node as an anatomical reference point to generate two regions corresponding approximately to dorsolumbar (top) and inguinal (bottom) sections (Figure 4B). Under thermoneutral conditions, heat production was similar between regions, consistent with low basal thermogenic activity. Following cold exposure, the inguinal (bottom) region exhibited significantly higher heat production compared with the dorsolumbar (top) region (Figure 4C,D). Consistent with these findings, qPCR analysis revealed increased *Ucp1* expression in the inguinal region relative to the dorsolumbar region after cold exposure (Figure 4E). Together, these results demonstrate that regional differences in thermogenic heat production within the iWAT depot are not evident under thermoneutral conditions but emerge following cold-induced thermogenic activation.

### 3.5. Effects of Tissue Storage Conditions on Thermogenic Heat Production and Adrenergic Responsiveness in Intact Adipose Explants

**Figure 5:**
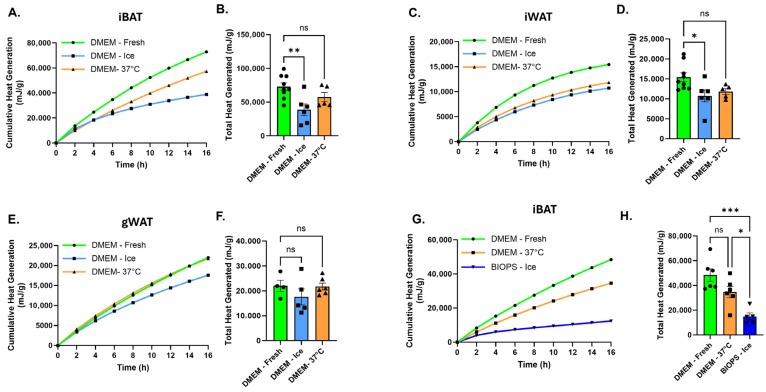
Effects of tissue storage conditions on thermogenic heat production in intact adipose tissue explants. (**A**,**B**) Cumulative heat production over time (**A**) and total heat production (**B**), normalized per gram of tissue, from iBAT explants (*n* = 9) that were freshly isolated or stored for 6 h in DMEM at 37 °C (*n* = 6) or on ice (*n* = 5) prior to isothermal microcalorimetry. (**C**,**D**) Cumulative heat production (**C**) and total heat production (**D**), normalized per gram of tissue, from iWAT explants under the same storage conditions. (**E**,**F**) Cumulative heat production (**E**) and total heat production (**F**), normalized per gram of tissue, from gWAT explants under the same conditions. (**G**,**H**) Cumulative heat production (**G**) and total heat production (**H**), normalized per gram of tissue, from iBAT explants that were freshly isolated (*n* = 6) or stored for 6 h in DMEM at 37 °C (*n* = 6) or in BIOPS buffer on ice (*n* = 5) prior to analysis; all samples were assayed in DMEM during the CalScreener run. Data are shown as mean ± SEM. Individual data points represent independent tissue explants. Statistical analyses were performed using one-way ANOVA as indicated. Tissue mass per well ranged from approximately 4–22 mg for iBAT and 40–175 mg for white adipose depots. Significance levels are indicated as follows: ns, not significant; * *p* < 0.05, ** *p* < 0.01, *** *p* < 0.001. Line colors and data point symbols in the cumulative heat generation plots correspond to those used in the adjacent total heat generation bar graphs.

Many metabolic studies require the harvest and analysis of adipose tissues from large cohorts of mice, necessitating extended tissue collection times. To assess whether short-term storage prior to microcalorimetric analysis affects thermogenic heat production, freshly harvested tissues were stored for up to 6 h either in nutrient-containing DMEM at 37 °C or on ice and compared with freshly isolated tissue loaded immediately into the CalScreener. All samples were loaded simultaneously to enable direct comparison of heat production. Storage in DMEM at 37 °C did not significantly impair thermogenic heat production in iBAT or iWAT relative to freshly isolated controls, whereas storage on ice significantly reduced heat output (Figure 5A–D). gWAT exhibited greater variability but showed a similar trend toward reduced heat production following cold storage (Figure 5E,F). Because BIOPS (biopsy preservation solution) is commonly used to preserve mitochondrial integrity and respiratory capacity during cold handling, we next tested whether storage of mitochondria-rich iBAT in BIOPS on ice maintains thermogenic heat production. Contrary to expectations, tissue stored in BIOPS on ice for 6 h exhibited reduced heat production compared with tissue maintained in DMEM, despite all samples being assayed in DMEM during microcalorimetric measurement (Figure 5G,H). Together, these results indicate that storage in nutrient-containing medium at physiological temperature preserves thermogenic heat production, whereas cold storage impairs calorimetric heat output in intact adipose tissue explants, even under conditions intended to preserve mitochondrial function, which was not directly assessed in this study.

**Figure 6:**
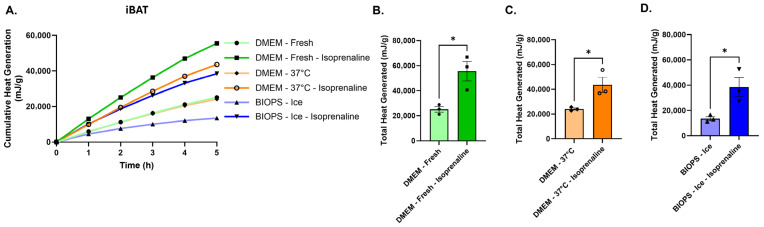
Preservation of β-adrenergic responsiveness following short-term storage of interscapular brown adipose tissue. (**A**) Cumulative heat production over time, normalized per gram of tissue, from iBAT explants that were freshly isolated or stored for 6 h in DMEM at 37 °C or in BIOPS buffer on ice, and subsequently treated with vehicle or the pan β-adrenergic agonist isoproterenol (**B**–**D**) Total heat production per gram of tissue from iBAT explants treated with vehicle or isoproterenol following fresh isolation (**B**), storage in DMEM at 37 °C for 6 h (**C**), or storage in BIOPS on ice for 6 h (**D**). Data are shown as mean ± SEM. Individual data points represent independent tissue explants (*n* = 3). Statistical analyses were performed using an unpaired Student’s *t*-test for within-condition comparisons. Between-condition comparisons of isoproterenol-treated groups were assessed by one-way ANOVA with Tukey’s post hoc test and showed no significant differences. Significance levels are indicated as * *p* < 0.05. Line colors and data point symbols in the cumulative heat generation plots correspond to those used in the adjacent total heat generation bar graphs.

Finally, we tested the ability of iBAT explants to respond to the pan β-adrenergic agonist isoproterenol under three conditions: freshly isolated iBAT, iBAT stored for 6 h in DMEM at 37 °C, and iBAT stored for 6 h in BIOPS on ice (Figure 6). Basal heat production was approximately two-fold higher in fresh tissue and in iBAT stored in DMEM at 37 °C compared with tissue stored in BIOPS on ice (Figure 6A–D). Despite these differences in baseline heat production, isoproterenol significantly increased heat output under all conditions. Fresh iBAT and iBAT stored in DMEM at 37 °C for 6 h exhibited similar increases in heat production of approximately two-fold relative to baseline. In contrast, iBAT stored in BIOPS on ice displayed a larger relative increase of approximately three-fold, restoring absolute heat production to levels comparable to those observed in isoproterenol-treated tissue stored in DMEM. Additional between-condition analysis of isoproterenol-treated samples showed no significant differences among freshly isolated iBAT, iBAT stored in DMEM at 37 °C, and iBAT stored in BIOPS on ice. Together, these results indicate that although cold storage reduces basal heat production, iBAT explants retain the capacity to mount robust β-adrenergic-induced increases in heat output.

## 4. Discussion

In this study, we apply isothermal microcalorimetry using the CalScreener as a platform for quantifying thermogenic heat production across a spectrum of adipose models, ranging from three-dimensional adipocyte spheroids to freshly isolated adipocytes and intact adipose tissue explants. Together, these data define key experimental parameters, clarify technical considerations, and provide practical guidance for the application of microcalorimetry to studies of adipose thermogenesis.

Using three-dimensional brown and beige adipocyte spheroids, we demonstrate that calorimetric heat output scales predictably with cell number across a defined range, with a plateau and decline at larger spheroid sizes. This behavior is consistent with diffusion constraints and functional limitations described in spheroid systems [20,21]. Importantly, pooling multiple spheroids per well improved measurement consistency, supporting multi-spheroid loading strategies for reproducible calorimetric measurements. These findings establish adipocyte spheroids as a tractable and scalable system for calorimetric interrogation of thermogenic function.

Although pH, nutrient availability (e.g., glucose, pyruvate, glutamine), and oxygen concentration were not directly measured at the end of the calorimetric experiments, several observations suggest that depletion of these factors is unlikely to fully explain the plateau in heat production observed at higher spheroid sizes. Reduced heat output in larger spheroids was evident early during the measurement period, prior to substantial medium depletion, and heat production increased approximately linearly over the 6 h assay across all conditions. In addition, morphological analysis revealed reduced cellular density and loss of adipocyte markers within the cores of larger spheroids, consistent with diffusion limitations in three-dimensional systems. Together, these findings support the interpretation that the observed plateau reflects structural and diffusion constraints rather than acute exhaustion of nutrients in the assay medium.

Extending these approaches to ex vivo preparations, we show that mature adipocytes freshly isolated from brown and subcutaneous adipose tissue remain viable and thermogenically responsive, mounting sustained heat production in response to β3-adrenergic stimulation. Notably, intact adipose tissue explants retained measurable thermogenic heat production following extended ex vivo handling when maintained in nutrient-containing medium at physiological temperature. These findings demonstrate that functional thermogenesis persists over experimentally relevant timeframes compatible with prolonged tissue harvests and batch microcalorimetric analysis.

Consistent with known in vivo physiology, intact adipose tissue explants exhibited depot-specific differences in thermogenic heat production, with brown adipose tissue displaying the highest basal heat output and thermoneutral housing markedly suppressing thermogenesis across depots. Brown adipose tissue exhibited the greatest reduction following thermoneutral housing, reflecting its strong dependence on sympathetic activation, whereas white adipose depots showed more modest changes. These findings indicate that isothermal microcalorimetry captures physiologically relevant modulation of thermogenic state in intact tissues ex vivo.

Prolonged microcalorimetric measurements revealed additional depot-specific differences in the temporal dynamics of heat production. Despite the well-established browning potential of inguinal adipose tissue in vivo, gonadal adipose tissue exhibited comparable or greater sustained heat production over extended measurement periods. This behavior was accompanied by distinct kinetic profiles, with inguinal adipose tissue displaying an early plateau in heat output, whereas gonadal adipose tissue maintained a sustained increase over time. These findings suggest that differences in the ability to sustain metabolic activity under ex vivo conditions, rather than intrinsic thermogenic capacity alone, may contribute to depot-specific differences in cumulative heat production. This interpretation is further supported by the divergence observed between thermogenic gene expression and calorimetric heat output. While *Ucp1* expression was more strongly induced in inguinal adipose tissue following cold exposure, this did not correspond to greater heat production under the conditions tested. Notably, full browning of inguinal adipose tissue typically requires prolonged cold exposure (approximately 7–10 days), raising the possibility that the 3-day cold challenge used here may not fully capture the maximal thermogenic capacity of this depot [24]. Together, these findings highlight that calorimetric heat production reflects an integrated functional output that may not be fully predicted by canonical thermogenic gene expression alone.

A key practical outcome of this work is the systematic evaluation of tissue storage conditions prior to isothermal microcalorimetry. We demonstrate that short-term storage of adipose tissue for up to 6 h in nutrient-containing DMEM at physiological temperature preserves thermogenic capacity comparably to freshly isolated tissue, enabling synchronized analysis of large experimental cohorts. In contrast, cold storage on ice markedly suppresses basal heat production. BIOPS is a physiological buffer widely used to preserve mitochondrial structure and respiratory capacity during ex vivo handling, owing to its defined ionic composition, inclusion of ATP and phosphocreatine, and buffering of calcium, which together help stabilize mitochondrial function during short-term cold storage, as demonstrated in prior studies [27,28]. However, BIOPS lacks exogenous metabolic substrates and is optimized for maintaining mitochondrial competence rather than supporting active cellular metabolism. Importantly, following storage, all tissues, including those stored in BIOPS, were returned to nutrient-containing DMEM for isothermal microcalorimetry. Under these equivalent assay conditions, BIOPS-stored tissues nonetheless exhibited reduced basal thermogenic activity, suggesting that preservation of mitochondrial function, as inferred from the known properties of BIOPS, is not sufficient to maintain integrated thermogenic capacity in intact adipose tissue explants.

Importantly, despite reduced basal heat output following cold storage, β-adrenergic responsiveness was preserved, with explants exhibiting robust stimulation of heat production upon isoproterenol treatment. This indicates that while cold storage suppresses basal thermogenic activity, the capacity to mount adrenergic responses remains intact. Conceptually, these findings reinforce that thermogenesis measured by isothermal microcalorimetry reflects a coordinated, systems-level metabolic output that depends on intact cellular signaling, substrate availability, and mitochondrial function, rather than mitochondrial function alone.

### Limitations and Future Directions

Several limitations of this study should be acknowledged. First, while isothermal microcalorimetry provides a sensitive and integrative measure of heat production, it does not directly resolve the underlying metabolic pathways contributing to thermogenesis. Future studies combining microcalorimetry with molecular, biochemical, and imaging-based approaches will be important for linking heat output to specific thermogenic mechanisms, including UCP1-dependent and UCP1-independent pathways. Second, the sealed nature of the CalScreener wells may impose constraints on oxygen and nutrient availability during prolonged measurements, which could differentially affect adipose depots and contribute to the divergence in heat production observed over extended time courses. Incorporating defined substrate supplementation or limiting measurement duration may help reduce metabolic stress and further clarify depot-specific differences in thermogenic stability. Third, although we demonstrate preservation of thermogenic heat production following extended ex vivo handling, longer storage durations were not examined. Defining the temporal limits of functional thermogenesis, as well as identifying conditions that extend tissue viability without compromising metabolic activity, represents an important area for future methodological development. Finally, the experiments presented here were performed primarily in murine adipose tissue and immortalized cell lines. Extension of these approaches to primary human adipose tissue, human adipocyte spheroids, and disease-relevant models will be critical for translating isothermal microcalorimetry into broader metabolic and translational research applications.

## 5. Conclusions

Overall, this work establishes isothermal microcalorimetry as a sensitive and versatile approach for studying adipose thermogenesis across multiple experimental models while defining key technical parameters related to sample configuration, assay duration, and tissue handling. By demonstrating that intact adipose tissue retains functional thermogenic heat production following extended ex vivo handling, these findings support the use of microcalorimetry for scalable and physiologically relevant studies of adipose metabolism under both experimental and translational contexts.

## Data Availability

The original contributions presented in this study are included in the article/Supplementary Material. Further inquiries can be directed to the corresponding author.

## Supplementary Materials

The following supporting information can be downloaded at https://www.mdpi.com/article/10.3390/cells15070579/s1, Figure S1: Adipogenic gene expression analysis in brown and beige adipocyte spheroids; Figure S2: Immunostaining and H&E staining analysis of beige adipocyte spheroids; Figure S3: Depot-specific thermogenic heat production in gonadal and inguinal adipose tissue explants; Figure S4: Assessment of regional thermogenic heat production within inguinal adipose tissue.
