# Supplementary material for: Direct Measurement of Adipose Thermogenesis by Isothermal Microcalorimetry
Source: Cells. 2026 Mar 25;15(7):579. doi: 10.3390/cells15070579 (PMC13072264; doi:10.3390/cells15070579)
Supplement: Supplementary file 1 [file cells-15-00579-s001.zip › cells-4121945-supplementary.pdf]

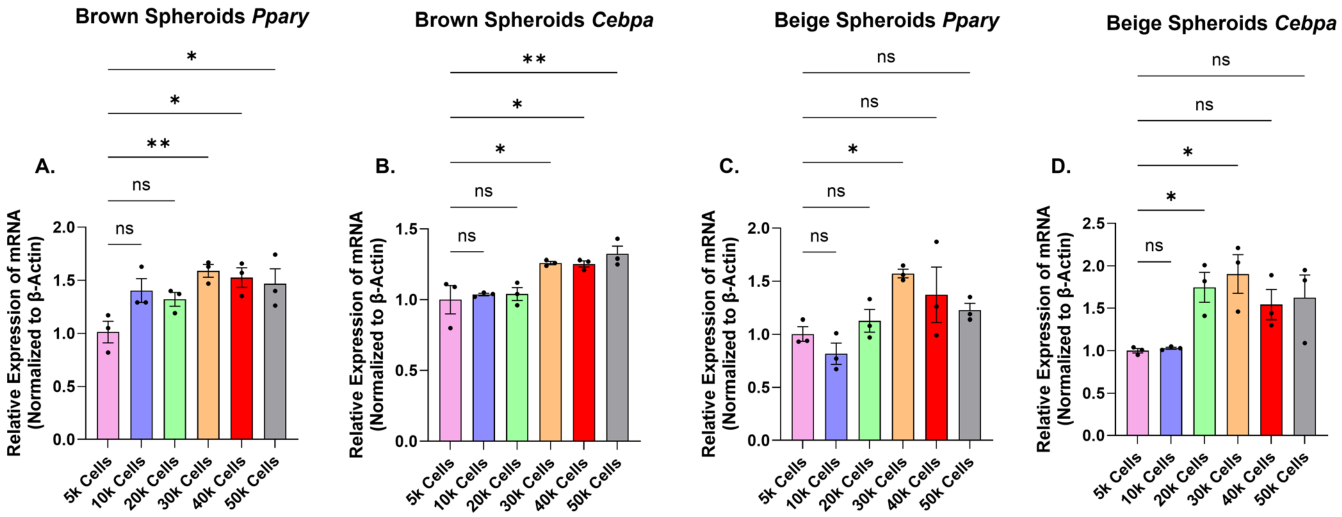

Supplementary Figure S1. Adipogenic gene expression analysis in brown and beige adipocyte spheroids. **(A)** qPCR analysis of *Pparγ* expression in brown adipocyte spheroids generated from 5,000-50,000 cells per spheroid (N = 3). **(B)** qPCR analysis of *Cebpa* expression in brown adipocyte spheroids generated from 5,000-50,000 cells per spheroid (N = 3). **(C)** qPCR analysis of *Pparγ* expression in beige adipocyte spheroids generated from 5,000-50,000 cells per spheroid (N = 3). **(D)** qPCR analysis of *Cebpa* expression in beige adipocyte spheroids generated from 5,000-50,000 cells per spheroid (N = 3). Data are shown as mean ± SEM. Individual data points represent independent qPCR measurements. Statistical analyses were performed using one-way ANOVA as indicated. Significance levels are indicated as follows: ns, not significant; * $p < 0.05$, ** $p < 0.01$.

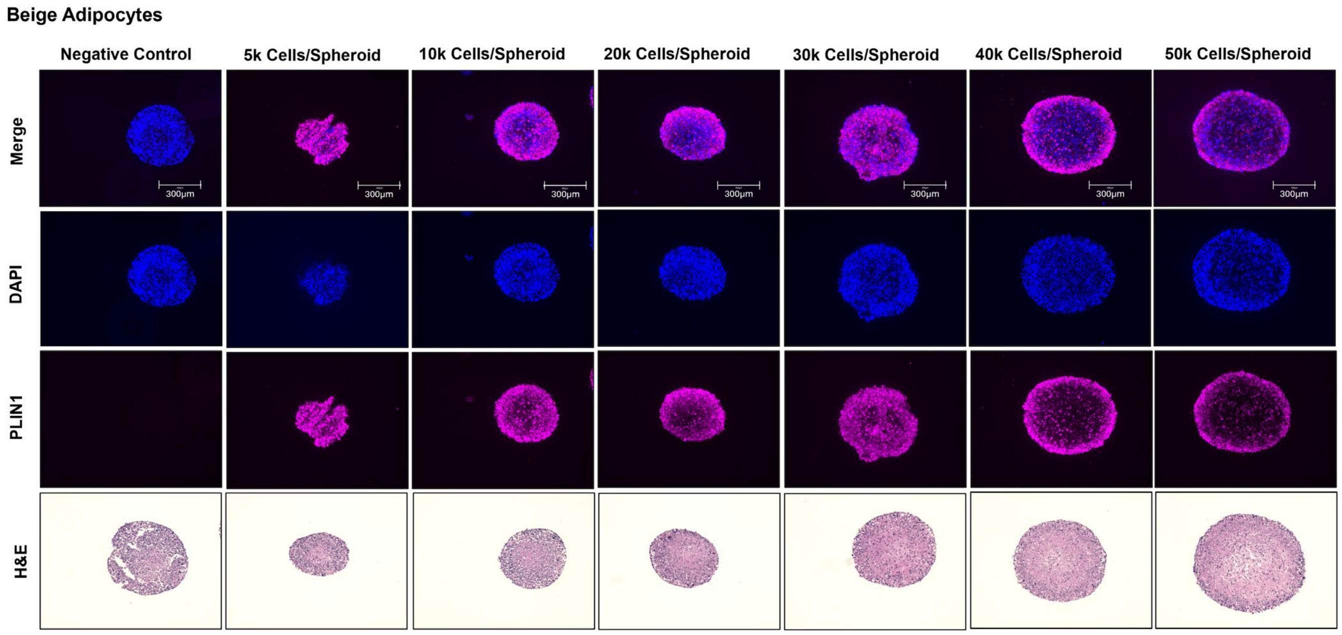

Supplementary Figure S2. Immunostaining and H&E staining analysis of beige adipocyte spheroids. Beige adipocyte spheroids were generated from preadipocytes seeded at 5,000-50,000 cells per spheroid. The top panels show representative cross sections of spheroids stained for PLIN1 (lipid droplets) and DAPI (nuclei). The bottom panels show corresponding hematoxylin and eosin (H&E) staining of spheroid sections. Spheroid diameter ranged approximately from 300 to 600 µm depending on the initial seeding density, as indicated by the scale bars.

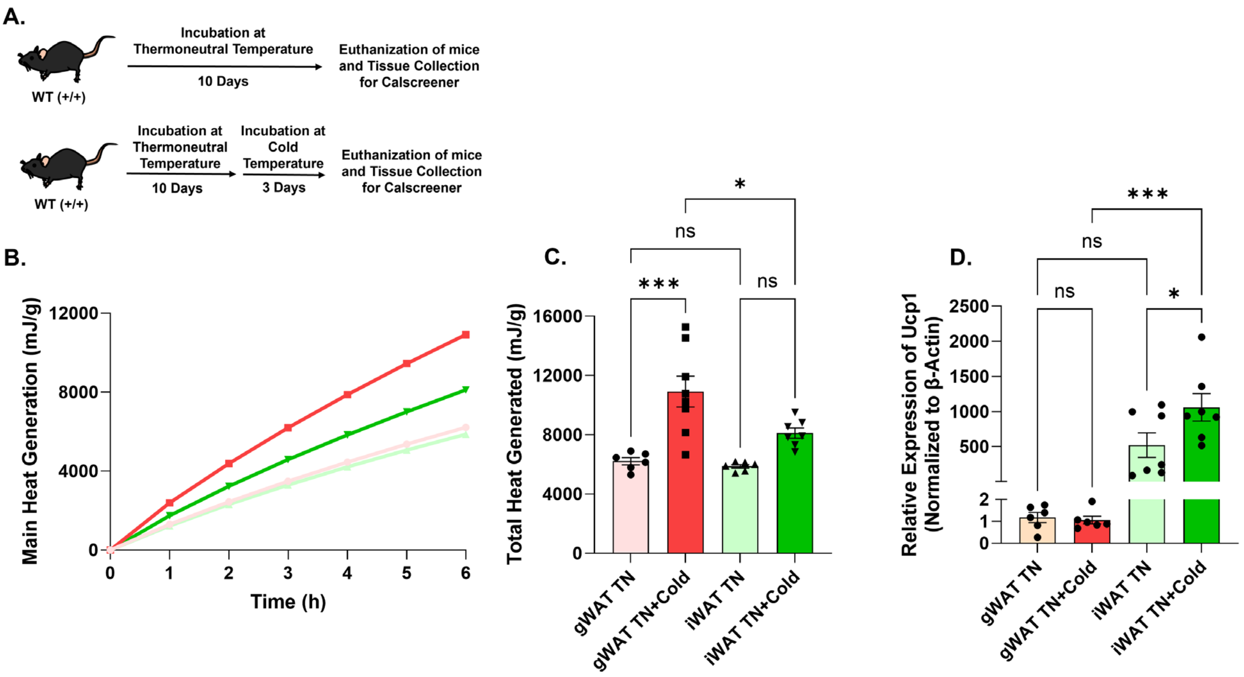

Supplementary Figure S3. Depot-specific thermogenic heat production in gonadal and inguinal adipose tissue explants. **(A)** Schematic illustration of wild-type mice housed under thermoneutral (TN) and cold (TN + Cold) conditions. **(B)** Cumulative heat production over time from iWAT and gWAT explants harvested from mice under each condition. **(C)** Total heat production per gram of tissue from iWAT and gWAT explants under thermoneutral and cold conditions (N = 6-8). **(D)** qPCR analysis of *Ucp1* expression in iWAT and gWAT explants under each condition (N = 6-7). Data are shown as mean ± SEM. Individual data points represent independent tissue explants **(C)** or independent qPCR measurements **(D)**. Statistical analyses were performed using one-way ANOVA as indicated. Significance levels are indicated as follows: ns, not significant; * $p < 0.05$, *** $p < 0.001$. Line colors and data point symbols in the cumulative heat generation plot correspond to those used in the adjacent total heat generation bar graph.

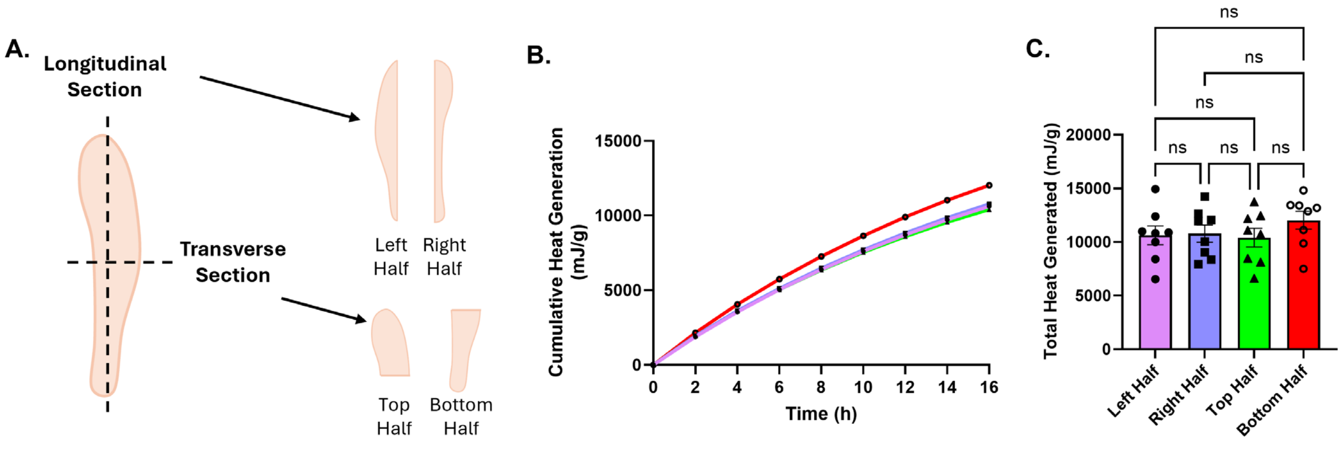

Supplementary Figure S4. Assessment of regional thermogenic heat production within inguinal adipose tissue. **(A)** Schematic illustrating longitudinal and transverse sectioning of the iWAT depot to generate anatomically distinct subsections. **(B)** Cumulative heat production over time, normalized per gram of tissue, measured by isothermal microcalorimetry from *ex vivo* iWAT subsections corresponding to left and right longitudinal halves and upper and lower transverse halves. **(C)** Total heat production per gram of tissue from each iWAT subsection (N = 8). Data are shown as mean ± SEM. Individual data points represent independent tissue sections. Statistical

analyses were performed using one-way ANOVA as indicated. Significance levels are indicated as follows: ns, not significant. Line colors and data point symbols in the cumulative heat generation plot correspond to those used in the adjacent total heat generation bar graph.
